# Genome Analysis of “*Candidatus* Regiella insecticola” Strain TUt, Facultative Bacterial Symbiont of the Pea Aphid *Acyrthosiphon pisum*

**DOI:** 10.1128/MRA.00598-20

**Published:** 2020-10-01

**Authors:** Naruo Nikoh, Tsutomu Tsuchida, Ryuichi Koga, Kenshiro Oshima, Masahira Hattori, Takema Fukatsu

**Affiliations:** a Department of Liberal Arts, The Open University of Japan, Chiba, Japan; b Bioproduction Research Institute, National Institute of Advanced Industrial Science and Technology, Tsukuba, Japan; c Faculty of Science, Academic Assembly, University of Toyama, Toyama, Japan; d Center for Omics and Bioinformatics, Graduate School of Frontier Sciences, University of Tokyo, Kashiwa, Japan; e Department of Biological Sciences, Graduate School of Science, University of Tokyo, Tokyo, Japan; f Graduate School of Life and Environmental Sciences, University of Tsukuba, Tsukuba, Japan; Indiana University, Bloomington

## Abstract

The genome of “*Candidatus* Regiella insecticola” strain TUt, a facultative bacterial symbiont of the pea aphid *Acyrthosiphon pisum*, was analyzed. We determined a 2.5-Mb draft genome consisting of 14 contigs; this will contribute to the understanding of the symbiont, which underpins various ecologically adaptive traits of the host insect.

## ANNOUNCEMENT

The species “*Candidatus* Regiella insecticola” is a bacterial clade of facultative symbionts that is associated with the pea aphid Acyrthosiphon pisum and other aphid species and belongs to the *Enterobacteriaceae* family of gammaproteobacteria (1). The symbiont is universally found in the world’s populations of A. pisum (2–5). Within the aphid body, the symbiont is localized to secondary bacteriocytes, sheath cells, and hemolymph (1, 6). Previous studies revealed that the symbiont is involved in a variety of context-dependent fitness consequences of the host aphid, including resistance to pathogenic fungi (7, 8), resistance to parasitoid wasps (9), adaptation to food plants (10–12), and others.

Here, we analyzed the genome of “*Ca.* Regiella insecticola” strain TUt, a facultative symbiont of A. pisum that was reported to influence food plant utilization of the host insect (10, 12). We collected body fluid of A. pisum strain AIST^TUt^, which was generated by artificial infection of the symbiont from its original host aphid strain (10, 12). Surface-sterilized adult aphids were dissected and washed in phosphate-buffered saline, the fluid was collected and filtered through 100-μm, 50-μm, and 10-μm nylon meshes, and the filtrate was subjected to DNA preparation with a standard phenol-chloroform method. The DNA sample (around 3 μg) was sheared to generate DNA fragments (2 to 4 kb), ligated to the pUC18 vector for shotgun library construction, and subjected to Sanger sequencing of both ends of the inserted fragments using an ABI 3730xl genetic analyzer with a read length of 1,000 bases, as described previously (13, 14). We obtained 26,215 sequence reads, of which 230 reads accounted for aphid genes (15) and 75 reads represented Buchnera aphidicola genes (16). The remaining 25,910 reads were subjected to assembly using the Phred v.0.020425.c-Phrap v.1.080812-Consed v.29.0 package with default parameters (17). Assembly gaps were closed by primer walking along the inserts and the PCR products containing the gaps. The genome assembly yielded 271 contigs with a total length of 2.67 Mb and an *N*_50_ value of 335,627 bp; 257 contigs were removed due to small size (0.2 to 2.4 kb) and insufficient sequence depth (1× to 6×). A sequence homology search indicated that these contigs were derived from contaminating DNA or repeated sequences, and no plasmid sequence was observed. The remaining 14 contigs represent the genome of “*Ca.* Regiella insecticola” strain TUt, which consisted of 25,316 reads with 10× coverage. The total size of the contigs (2,495,260 bp, with a GC content of 42.4%) was almost equivalent to the size of the draft genome sequence of “*Ca.* Regiella insecticola” strain LSR1 (2,110,331 bp, with a GC content of 40.1%) (18). Strain LSR1 was reported to contain a 32.5-kb plasmid (18), while it is not known whether strain TUt also possesses a plasmid. We assessed the quality of the finished sequence by the Phred score (≥40). Putative protein-coding sequences (CDSs), tRNAs, and other noncoding RNAs were identified using GLIMMER v.3.0 (19) and Prokka v.1.14.5 (20). The annotation of CDSs was based on homology searches against UniProt (21). In the draft genome sequence of “*Ca.* Regiella insecticola” strain TUt, we identified 2,443 putative protein-coding genes (of which 723 were located within repetitive sequences as transposases), 11 rRNA genes, 43 tRNA genes, and 408 pseudogenes.

To date, genome sequences of “*Ca*. Regiella insecticola” have been analyzed for another A. pisum-associated strain, LSR1 (18), and strain 5.15 from the green peach aphid Myzus persicae, which causes resistance to parasitoid wasps (22). These genome sequences of “*Ca*. Regiella insecticola” will enable comparative genomic analyses for understanding the mechanisms of the symbiont-mediated ecological adaptations.

### Data availability.

The genome sequence of “*Ca*. Regiella insecticola” strain TUt has been deposited in the DNA Data Bank of Japan (DDBJ) under accession no. BLXO01000001 to BLXO01000014 (draft genome) and DRA009060 (raw sequence reads).
